# Study on the clinical assessment of integrated rehabilitation of Traditional Chinese Medicine and western medication for acute myocardial infarction

**DOI:** 10.1097/MD.0000000000021592

**Published:** 2020-08-21

**Authors:** Zhiqiang Zhao, Xianliang Wang, Shuai Wang, Ruijuan Zhou, Yu Liu, Lishuo Su, Chenyu Li, Shanshan Lin, Hua Liu, Lindan Zhao, Jingyuan Mao

**Affiliations:** Cardiovascular Department, First Teaching Hospital of Tianjin University of Traditional Chinese Medicine, Tianjin, China.

**Keywords:** Baduanjin, myocardial infarction, peak oxygen consumption, protocol, randomized controlled trial, rehabilitation

## Abstract

**Objective:**

This trial aims to evaluate the efficacy and safety of the Baduanjin exercise in patients with acute myocardial infarction (AMI).

**Methods:**

A single-center, open, randomized controlled clinical trial will be conducted to evaluate the effectiveness of the Baduanjin exercise on the rehabilitation of AMI patients. It plans to enroll 64 patients. Patients will be divided evenly into 2 groups using a random number table method. There will be 32 cases in each group. Patients in the experimental group will be treated with standardized drug therapy combined with Baduanjin exercise, while patients in the control group will be treated with standardized drug therapy combined with routine exercise. The primary outcome is the peak oxygen consumption (Peak VO_2_) during cardiopulmonary exercise test (CPET). The secondary outcomes include CPET, echocardiography, Seattle angina pectoris scale, hospital depression and anxiety scale, Pittsburgh Sleep Quality Index scale, scores of 4 examinations, and diagnostic methods of traditional Chinese medicine and composite endpoint events, etc.

**Discussion:**

This study will be the first to evaluate the effect of the Baduanjin exercise on the Peak VO_2_ in patients with AMI.

**Study registration:**

This study has been registered on the Chinese Clinical Trial Registry (No: ChiCTR1800016209, protocol version 1.2).

## Abstract 2

Supplemental Digital Content is available in the text

## 1 Introduction

The incidence of acute myocardial infarction (AMI) is increasing year by year in China.^[1]^ Revascularization and drug therapy could improve the symptoms and reduce the mortality rate of AMI patients.^[2,3]^ However, survivors following reperfusion therapy still have some problems, such as reduced exercise tolerance, poor quality of life, anxiety, depression, etc. The mortality rate, the risk of incident stroke, or rehospitalization rate within the first 6 months after discharge from the hospital is about 25%.^[4]^ Some studies have shown that aerobic exercise in cardiac rehabilitation can improve exercise ability, reduce the all-cause mortality, cardiovascular mortality, and rehospitalization rate of AMI patients.^[5–7]^ However, only 38.8% of countries in the world have a cardiac rehabilitation program.^[8]^ Participating in the cardiac rehabilitation program may be restricted due to aging, solitude, absence of awareness, no medical insurance, etc.^[9]^ Alternative simple and feasible exercise-based rehabilitation for AMI will still be needed.

Tai Chi and Yoga exercise is economical and time-saving. However, they include some complex actions and irregular practices can cause sports injuries.^[10]^ The use of these exercises is restricted in the early stage of AMI. Baduanjin exercise is a low-intensity aerobic exercise with simple actions and easy to learn.^[11,12]^ Some studies have shown that Baduanjin exercise can improve lung function, exercise ability, control blood pressure, blood lipid, quality of life, quality of sleep, relieve depression, and other adverse emotions.^[13–16]^ There is no clinical study on the effect of the Baduanjin exercise on exercise tolerance in patients with AMI.

## 2 Methods

### 2.1 Study design

It is a single-center, open, randomized controlled clinical trial. We will enroll 64 patients with AMI in the Cardiovascular Department of the First Teaching Hospital of Tianjin University of Traditional Chinese Medicine. The informed consent was approved by the Ethics Committee of First Teaching Hospital of Tianjin University of Traditional Chinese Medicine (TYLL2018[K]012). It was registered in the Chinese clinical trial registry (ChiCTR1800016209). The Standard Protocol Items: Recommendations for Interventional Trials (SPIRIT) checklist is provided as Supplemental Digital Content (Additional file 1).

### 2.2 Inclusion criteria

1. Patients were ST-elevation myocardial infarction or non-ST-elevation myocardial infarction confirmed by coronary angiography and treated with reperfusion therapy, whose condition became relatively stable after treatment.
2. Aged more than 30 years old;
3. Willing to participate in cardiac rehabilitation;
4. Sign up the informed consent.

### 2.3 Exclusion criteria

1. Patients with severe cardiovascular diseases such as unstable angina, uncontrolled heart failure, malignant arrhythmia, cardiac shock, uncontrolled hypertension (systolic pressure more than 160 mm Hg, diastolic pressure more than 100 mm Hg), severe ventricular/supraventricular arrhythmia, uncontrolled tachycardia (heart rate >120 bpm in a resting state), and severe valvular heart valve disease that requires surgical intervention, hypertrophic obstructive cardiomyopathy, acute pericarditis, congenital heart disease, etc.
2. Patients with severe pulmonary disease, uncontrolled diabetes, severe hepatic and renal insufficiency (creatinine < 30 mL/min), malignant tumor, and other diseases;
3. Severe conditions as severe myocardial ischemia or hypoglycemia occurred during the exercise;
4. Patients with neurological or orthopedics diseases, or with dementia/cognitive impairment and other related diseases, who cannot carry out rehabilitation exercise training;
5. Patients with poor compliance or who refuse to sign up the informed contest;
6. Patients who have practiced Baduanjin or Taichi in the past;
7. Other situations that are not suitable for participation in trials.

### 2.4 Interventions

The standardized drug therapy is recommended by Guidelines for Diagnosis and Treatment of Acute ST-elevation Myocardial Infarction^[17]^ and Guidelines for Diagnosis and Treatment of Non-ST-elevation Acute Coronary Syndrome (2016).^[18]^

During hospitalization, experimental group patients change from sitting Baduanjin exercise to vertical Baduanjin exercise gradually under guardianship according to their condition.

When they were discharged from the hospital, they exercised 3 to 5 times a week, including 5 minutes of warm-up exercise (such as stretching and joint activity, etc), 10 to 15 minutes of Baduanjin exercise, and 5 minutes finishing exercise after Baduanjin exercise. The intervention measures of patients in the control group were standardized drug therapy combined with routine exercise (except traditional sports such as Baduanjin, Tai Chi Chuan, Yoga, etc). During hospitalization, the patients in the control group exercised under guardianship according to their condition. After discharge, the exercise prescription was made according to the results of peak oxygen consumption (CPET): self-feeling tired degree from easy to hard, 3 to 5 times every week, 30 minutes every time.

Patients in the 2 groups need to come to the hospital for outpatient follow-up and exercise and medication guidance every 2 weeks within 3 months after discharge. During 3 to 6 months after discharge, the researcher will encourage the patients to independently adhere to Baduanjin exercise or routine exercise by telephone/WeChat. The observation notes include the time of inclusion, enrollment (V0), discharged (V1), discharged from hospital for 1 month (28 ± 7) days (V2), discharged from hospital for 3 months (84 ± 7) days (V3), discharged from hospital for 6 months (168 ± 7) days (V4).

### 2.5 Outcomes

Primary outcome: peak oxygen consumption (Peak VO_2_) was the main outcome measure. Patients underwent a CPET at V1, V2, and V3.

Secondary outcomes: The power and other indicators of CPET will be recorded at V1, V2, and V3. The ultrasound testers investigate echocardiography for patients at V0, V1, V2, and V3. Seattle angina pectoris scale, Hospital Depression Anxiety Scale, Pittsburgh Sleep Quality Index scale (PSQI), Scores from the 4 examinations, and diagnostic methods of traditional Chinese medicine will be completed by the same trained physician. The composite endpoint is an aggregative indicator including all-cause death, AMI, revascularization, heart failure, malignant arrhythmias, cardiogenic shock, stroke, and pulmonary embolism. Statistical indicators include B-type natriuretic peptide (BNP), 2, 4, 6-trinitrotolurene (TnT); hypersensitive C-reactive protein (hs-CRP), 4 items of blood lipid, urinary albumin clearance rate, intravenous glucose, outpatient or hospitalization expenses, number of hospitalization, etc.

Safety outcomes: The safety outcomes include blood pressure, heart rate, 12-point resting electrocardiogram, blood routine, serum potassium, serum creatinine, urea nitrogen, and adverse events.

### 2.6 Follow-up time and measurement indexes

Baseline data includes the patient's current vital signs, medications taken, and coronary artery revascularization. Laboratory tests include blood pressure, BNP, TnT, hs-CRP, serum potassium, creatinine, urea nitrogen, intravenous glucose, 4 items of blood lipid, urinary albumin excretion rate.

**Figure 1:**
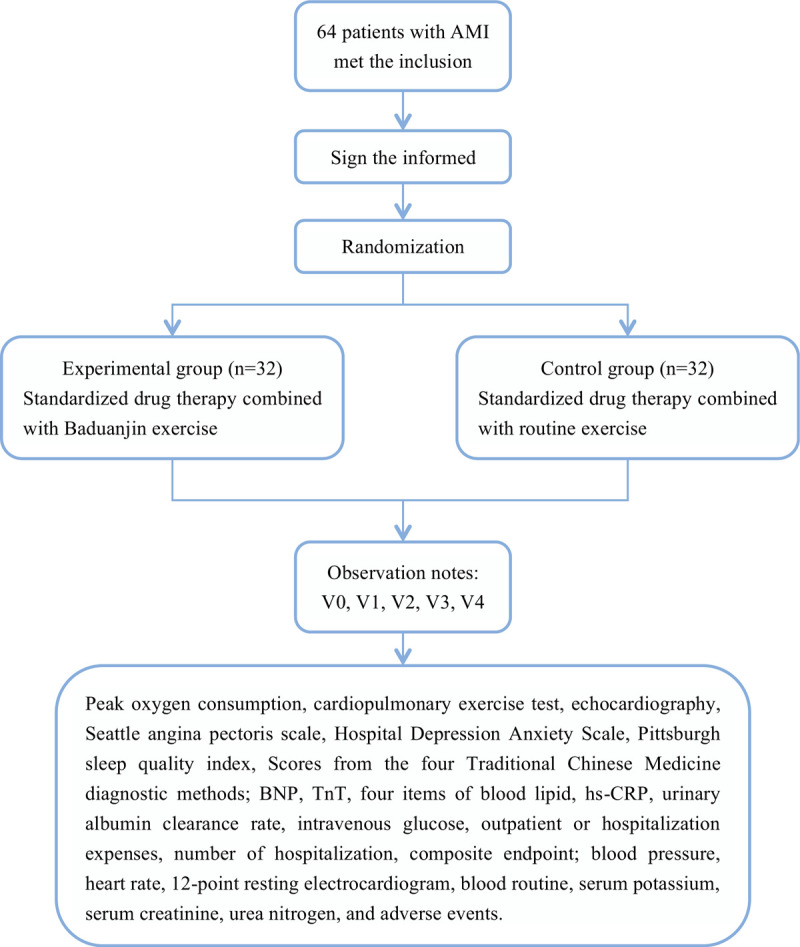
Flow chart of research implementation. Notes: AMI  = acute myocardial infarction, V0 = enrollment, V1 = discharged, V2 = discharged from hospital for 1 month (28 ± 7) days, V3 = discharged from hospital for 3 months (84 ± 7) days, V4 = discharged from hospital for 6 months (168 ± 7) days, BNP =  B-type natriuretic peptide, TnT = 2, 4, 6-trinitrotolurene, hs-CRP =  hypersensitive C-reactive protein.

After enrollment, patients are followed up in the hospital at V1, V2, and V3, and telephone follow-up at V4. Details are given in Table 1. The implementation process is shown in Figure 1.

**Table 1:**
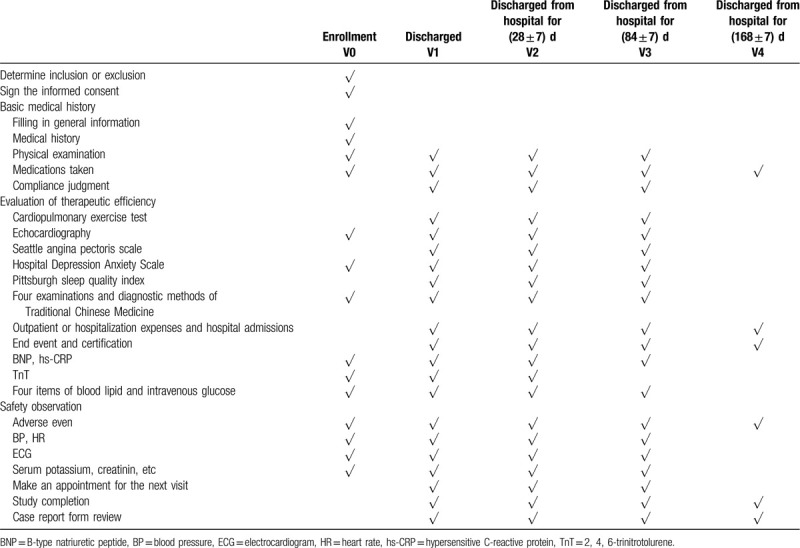
Summary table of research procedures.

### 2.7 Sample size

According to a previous trial,^[19]^ Peak VO_2_ at the end of the twelfth week of the treatment was 24.6 ± 5.2 mL/kg/min in the experimental group and 19.4 ± 4.4 mL/kg/min in the control group. The experimental group and the control group are grouped in a 1: 1 ratio. The significance level α is 0.05, and the power (1 − β) is 0.80. Considering an unfinished rate of no more than 20%, the number of cases in each group is at least 32. The calculation formula is listed as follows: 
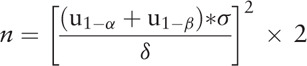


### 2.8 Randomization method and blinding

After the patient signs the informed consent, they will be assigned into the experimental group or control group by stratified randomization (1:1) according to whether the ST segment elevates or not. The random number table will be saved by a special person who will not participate in the assignment, specific implementation, data management, and statistical analysis. Laboratory personnel, ultrasound testers, CPET operators, and statistical analysts are unaware of patients’ grouping and interventions. And there is no blinding in the outcome assessment.

### 2.9 Data monitoring

A supervisor will be set up to monitor and evaluate the safety of patients. The supervisor uses this data to guide the implementation of the study, monitor clinical evaluations, conduct standardized guidance, and determine whether the trial should be terminated.

### 2.10 Data management and statistical analysis

An Excel data-sheet is created. An independent researcher will check the data and lock the database before statistical analysis. Statistical analysis is performed using SPSS 19.0. For counting data, frequency and percentage are used for data description, and the *χ*^2^ test is used for data comparison. For measurement data, the *t* test is used for normal distribution data. Interval data are presented with means and 95% confidence intervals. The rank-sum test is used for non-normal distribution data. *P* < .05 indicates that the difference is statistically significant. Participants who have received treatment but there is no valid evaluation data will be considered as missing and will be included in the effectiveness analysis.

## 3 Discussion

This trial aims to evaluate whether standardized drug therapy combined with Baduanjin exercise can improve exercise tolerance in patients with AMI.

Traditional exercises such as Tai Chi^[19,20]^ and yoga^[21,22]^ are beneficial for patients with cardiovascular disease. These exercises have no special requirements on the field and are therefore easily accepted by patients. Patients with AMI usually exercise Taijiquan 14 to 21 days after discharge.^[19]^ However, irregular exercises and insufficient leg strength are likely to cause knee joint pain, which often occurs in the initial learning stage.^[23]^ Some antijoint activities of yoga are beyond the range of normal joints, which can cause sports injuries,^[10]^ and then affect compliance. Baduanjin exercise is moderate in intensity and short in duration (a set of Baduanjin takes about 12 min). Compared to Tai Chi and Yoga, the Baduanjin is easier to learn. The vertical Baduanjin will help enhance the stability of the lower limbs.^[24–26]^ The exercise intensity of Baduanjin can be adjusted according to the patient's condition, which has certain advantages for cardiac rehabilitation of AMI.

Exercise capacity is an independent predictor of all-cause mortality and cardiovascular mortality in patients with AMI.^[27,28]^ CPET is used to test the exercise capacity of patients with AMI.^[29,30]^ Studies^[31]^ have shown that early exercise rehabilitation after AMI is beneficial, but 3 to 5 days after AMI is an absolute contraindication to CPET.^[32]^ Therefore, CPET is selected at the time of discharge in this study. As the primary outcome, Peak VO_2_ can reflect exercise capacity and predict long-term prognosis.^[33]^ Left ventricular function measured by echocardiography can predict cardiovascular events^[34]^ and cardiac workload^[35]^ in patients with AMI. BNP is an important indicator for predicting heart failure in patients with AMI.^[36]^ TnT and hs-CRP can reflect the severity of myocardial necrosis and are related to the prognosis.^[37]^ Seattle angina pectoris scale is used to evaluate the clinical symptoms and quality of life, Hospital Depression Anxiety Scale, and PSQI are used to reflect the internal mental state of patients. The composite endpoint was evaluated in 6 months after discharge. Currently, there is no randomized controlled trial about the effect of the Baduanjin exercise on exercise tolerance for patients with AMI. This study is expected to prove that Baduanjin exercise is a potentially valuable exercise rehabilitation method for patients with AMI.

The trial has some limitations. A single-center design may lead to selective bias in patient inclusion. Owing to the small sample size and many exclusion conditions, some high-risk patients who have not undergone revascularization or incomplete revascularization may be excluded, which may limit the extrapolation of conclusions. Nonmedical supervised training may affect patient compliance, making trial results subject to confounding factors. In conclusion, the trial aims to prove that combined with the Baduanjin exercise based on standardized drug treatment can improve exercise tolerance and long-term prognosis of patients with AMI after revascularization.

## Acknowledgments

The authors thank all the participants in their study.

## Author contributions

**Data collection:** Shuai Wang, Ruijuan Zhou, Yu Liu.

**Project administration:** Zhiqiang Zhao, Xianliang Wang.

**Recruitment patients:** Zhiqiang Zhao, Lishuo Su, Chenyu Li, Lindan Zhao.

**Trial design:** Jingyuan Mao, Zhiqiang Zhao, Xianliang Wang.

**Writing – original:** Zhiqiang Zhao, Xianliang Wang, Shanshan Lin, Hua Liu.

**Writing – review & editing:** Zhiqiang Zhao, Xianliang Wang, Jingyuan Mao.

## Supplementary Material

Supplemental Digital Content
